# Monolayer MoS_2_ field effect transistor with low Schottky barrier height with ferromagnetic metal contacts

**DOI:** 10.1038/s41598-019-53367-z

**Published:** 2019-11-19

**Authors:** Sachin Gupta, F. Rortais, R. Ohshima, Y. Ando, T. Endo, Y. Miyata, M. Shiraishi

**Affiliations:** 1 Department of Electronic Science and Engineering, Kyoto University, Kyoto, Kyoto, 615-8510 Japan; 2 Department of Physics, Tokyo Metropolitan University, Hachioji, 192-0397 Tokyo, Japan

**Keywords:** Materials science, Nanoscience and technology

## Abstract

Two-dimensional MoS_2_ has emerged as promising material for nanoelectronics and spintronics due to its exotic properties. However, high contact resistance at metal semiconductor MoS_2_ interface still remains an open issue. Here, we report electronic properties of field effect transistor devices using monolayer MoS_2_ channels and permalloy (Py) as ferromagnetic (FM) metal contacts. Monolayer MoS_2_ channels were directly grown on SiO_2_/Si substrate via chemical vapor deposition technique. The increase in current with back gate voltage (*V*_g_) shows the tunability of FET characteristics. The Schottky barrier height (SBH) estimated for Py/MoS_2_ contacts is found to be +28.8 meV (at *V*_g_ = 0V), which is the smallest value reported so-far for any direct metal (magnetic or non-magnetic)/monolayer MoS_2_ contact. With the application of positive gate voltage, SBH shows a reduction, which reveals ohmic behavior of Py/MoS_2_ contacts. Low SBH with controlled ohmic nature of FM contacts is a primary requirement for MoS_2_ based spintronics and therefore using directly grown MoS_2_ channels in the present study can pave a path towards high performance devices for large scale applications.

## Introduction

Two-dimensional (2D) materials with their layered structures have attracted much attention as next generation device materials due to their extraordinary properties such as mechanical flexibility, large surface to volume ratio, and their easy integration in heterostructure junction devices^1–3^. Graphene is well known example among these materials, which shows very rich physics resulting from its linear dispersion relation and massless Dirac Fermion^4^. Owing to remarkable properties such as very high mobility, large electrical and thermal conductivity, high Young’s modulus and small spin-orbit coupling (SOC), graphene became a promising candidate for wide range of applications, including high speed electronics, sensors, energy generation and storage devices as well as spintronics^5–7^. However, semi-metallic nature (gapless band structure) of pristine graphene limits its application in semiconductor electronics as zero band-gap leads a low on/off ratio in graphene-based field effect transistors (FETs)^4,8^. In addition to this, small SOC in graphene does not allow this material to have better control on generation and electrical manipulation of spins in spintronic devices.

Unlike graphene, molybdenum disulfide (MoS_2_), which belongs to the family of transition metal dichalcogenides (TMDs) shows semiconducting nature with a sizable band-gap^3^. The type and value of band-gap in MoS_2_ can be changed by varying the number of layers—MoS_2_ shows an indirect bandgap (~1.2 eV) in the bulk form (multilayers) and a direct band-gap (~1.8 eV) when reduced to monolayer^3^. In addition to non-zero band-gap, MoS_2_ also possesses considerable SOC along with unique spin-valley coupling to manipulate the spins, which makes the material very attractive for the next generation spintronic and other technological applications. The interest in mono-layer MoS_2_ has further increased after the demonstration of a high on/off ratio (~10^8^) and high carrier mobility at room temperature FETs^9,10^. However, the large electrical potential drop due to high contact resistance between MoS_2_ and metal contacts may strongly limit the performance of MoS_2_-based devices. Previous reports show large Schottky barrier heights (SBHs) for various metal/MoS_2_ contacts^11^, which can be reduced by different approaches such as insertion of insulating layers (*h*-BN^12^, MgO^13^, TiO_2_^14,15^, Al_2_O_3_^16^) between metal and MoS_2_, chemical doping^17,18^ of MoS_2_ and electrical gating^13,16^. To study spin injection from ferromagnetic (FM) metal and spin transport in MoS_2_, it is very important to investigate the contact behavior between FM metal and MoS_2_ and to suppress the SBH that hinders efficient spin injection/detection. There are only few reports in the literature, which discussed behavior of FM/monolayer MoS_2_ contacts and estimated SBH^12,13,16^. From our knowledge, the MoS_2_ in previous reports is either exfoliated from the bulk single crystal of MoS_2_ or transferred from MoS_2_ sample grown via chemical vapor deposition (CVD) technique. However, it is notable that these methods are not suitable for mass production of electronic devices and exfoliation and/or transfer methods can induce unwanted changes in physical and electronic properties of MoS_2_. In addition, the FET device characteristics strongly depend on the growth methods of MoS_2_ and FM electrodes. Hence, it will be interesting to study the behavior of FM/MoS_2_ contacts, where MoS_2_ is directly grown on substrate by CVD technique.

In this paper, we study the device characteristics of FETs fabricated using monolayer MoS_2_ channels, directly grown on SiO_2_/Si substrate using salt-assisted CVD technique. 20 nm thick Ni_80_Fe_20_ (Py) electrodes were used as ferromagnetic contacts. The work function of Py is 4.83 eV^19^. To understand contact behavior of Py/MoS_2_ contacts, *I*-*V* characteristics were studied with temperature and back gate voltages as controlling parameters. The SBH of Py/MoS_2_ contacts was determined to be +28.8 meV at the zero-gate voltage. Such contacts with low SBH and ohmic nature can play a key role in future spin-based devices because the tuning of the SBH allows circumventing the conductance mismatch problem for injecting spins in semiconductors^20^.

## Experimental Details

**Figure 1:**
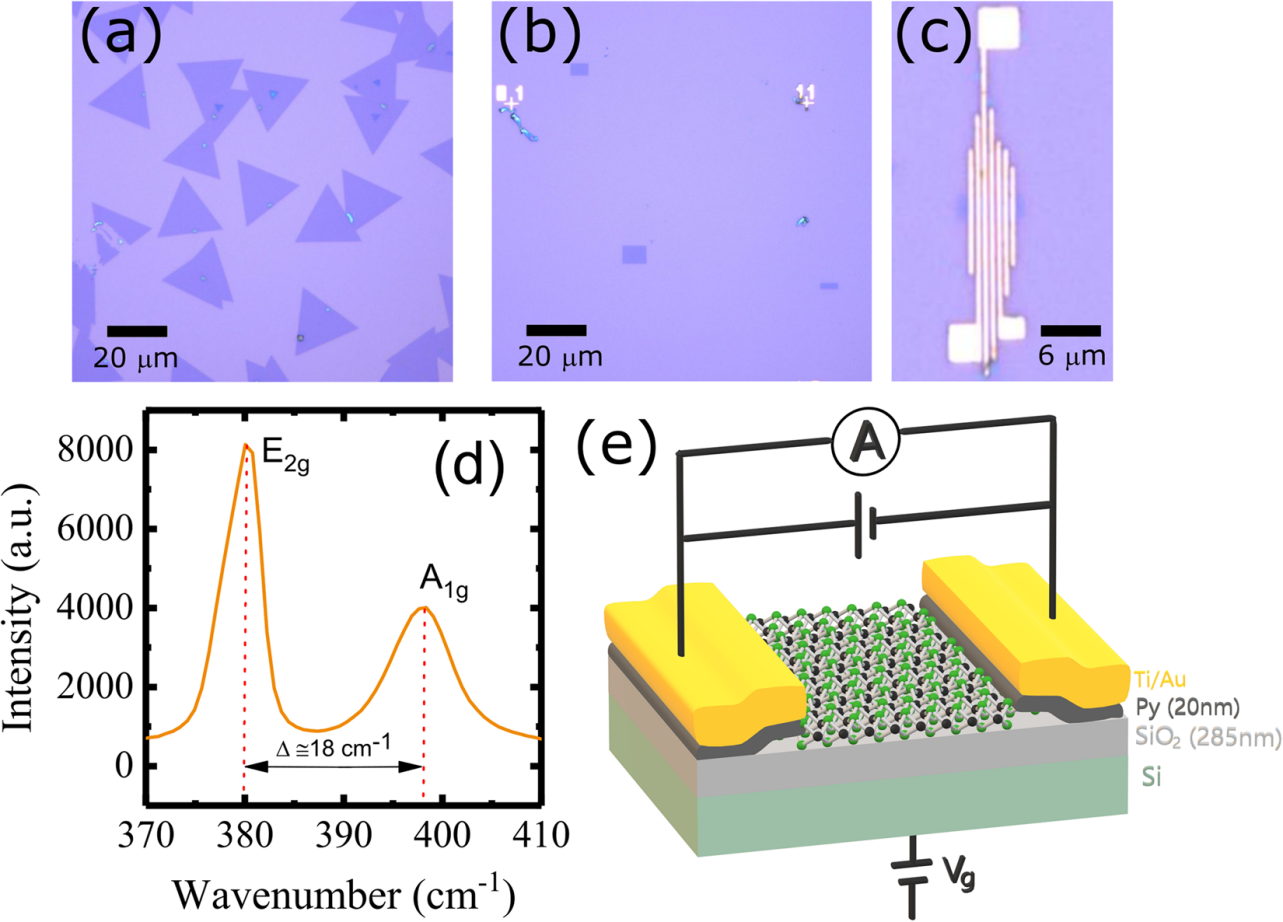
Optical microscope images of monolayer MoS_2_—(**a**) As grown MoS_2_ (triangular shaped), (**b**) MoS_2_ channels of different dimensions after processing (rectangular shaped) and (**c**) MoS_2_ device with permalloy (Py) electrodes. (**d**) Raman spectra of monolayer MoS_2_ performed with laser light of wavelength of 488 nm. (**e**) The schematic of a FET device and measurement configuration for two-probe source-drain current-voltage (*I*_DS_-*V*_DS_) curves.

The MoS_2_ is grown on the thermally oxidized SiO_2_/n^+^-Si substrate with SiO_2_ thickness of ~285 nm via salt-assisted CVD technique (please see ref.^21,22^ for the growth procedure). The monolayer crystal grown by this method were found to be almost free from defects/vacancies and any unintentional doping with alkali and/or halogen atoms^22^. Moreover, the sharp photoluminescence spectrum and the largest mobilities observed for these samples confirm its excellent crystal quality over exfoliated and CVD grown samples^22,23^. Figure 1(a) shows an optical microscope image of as-grown monolayer MoS_2_, which was processed in different channels shown in Fig. 1(b). Afterwards, Py electrodes of thickness 20 nm were deposited on the top of MoS_2_ channels, capped by Ti(3 nm)/Au(50 nm) as shown in Fig. 1(c). The monolayer MoS_2_ was confirmed by the Raman spectroscopy with a laser light of wavelength of 488 nm. Only monolayer MoS_2_ was processed for the fabrication of FET devices. Figure 1(d) shows Raman spectra for MoS_2_ samples. The two prominent peaks in the Raman spectra appear due to an in-plane (E_2g_) mode located around 382.2 cm^−1^ and an out-of-plane (A_1g_) mode located around 398.2 cm^−1^. The difference between these two modes is found to be ~18 cm^−1^, which confirms the monolayer MoS_2_^24^. The MoS_2_ channels [Fig. 1(b)] of length (*l*) 6–8 µm and width (*w*) 2–4 µm have been prepared by electron-beam (EB) lithography using TGMR resist (negative tone). In the first step, the MoS_2_ channels were covered by TGMR resist and unwanted MoS_2_ is etched by O_2_ plasma etching for 20 seconds. In the next step, TGMR resist was lifted by wet etching using N-Methyl-2-pyrrolidone and then cleaned by acetone and isopropyl alcohol (IPA). Py(20 nm)/Ti(3 nm)/Au(50 nm) electrodes were deposited by EB deposition technique after patterning them by EB lithography using Poly(methyl methacrylate) (PMMA) resist. The center-to-center distance between two electrodes was 0.65 µm. The electrical measurements have been performed using helium-free cryostat in the temperature range 10–300 K. The gate voltage was applied from the backside of the Si substrate.

## Results and Discussion

Figure 1(e) shows the schematic for FET device and measurement configuration. Source-drain current-voltage (*I*_DS_-*V*_DS_) characteristics were performed by applying DC voltage and recording the current between two probes. We measured five devices (device #A1, #A2, #B1, #B2 and #C1) from three different samples (A, B and C) to check the consistency of our experimental results. In the main text, we mainly discuss FET characteristics of device #A1 while results of other devices are used as supporting information and are shown in Supplementary Material.

**Figure 2:**
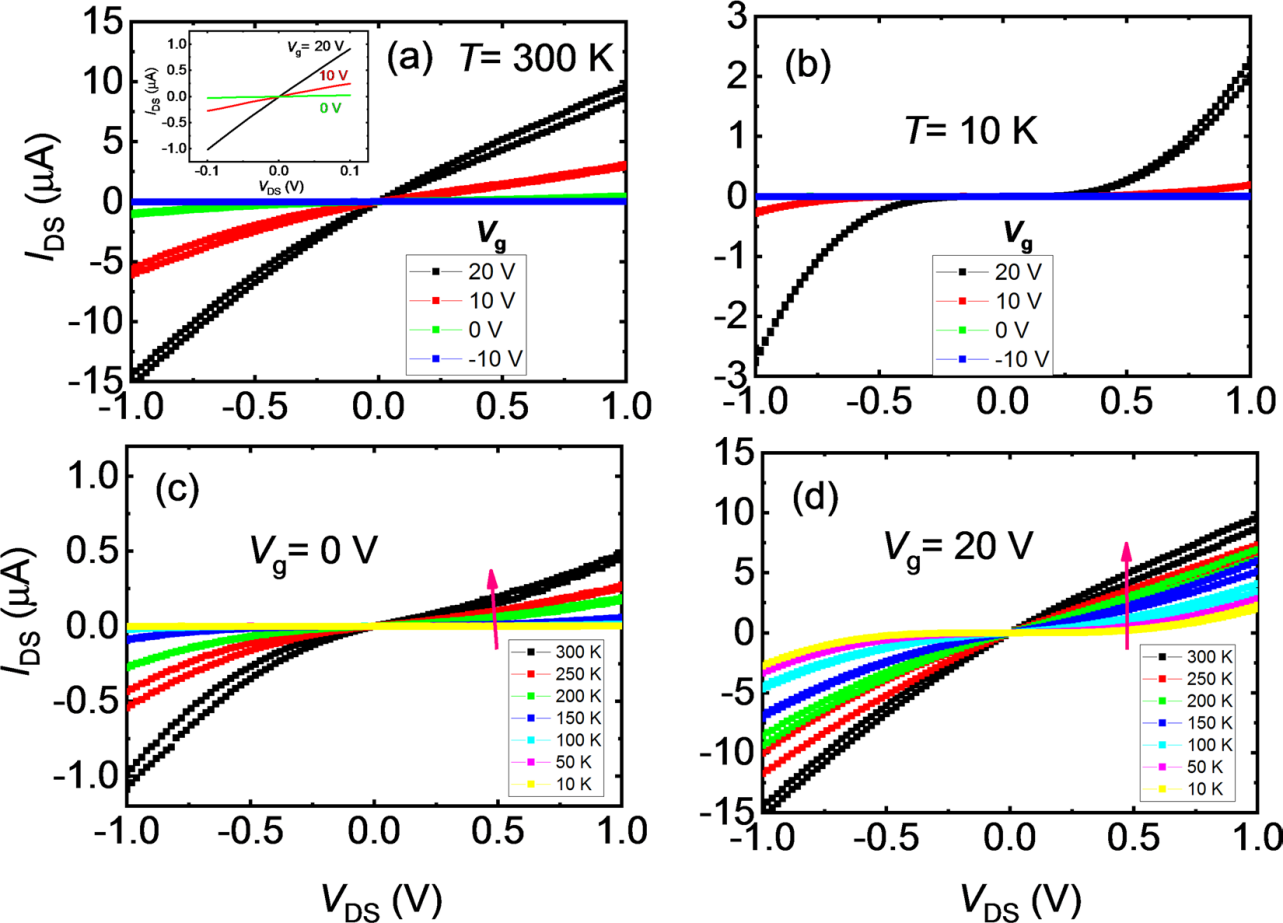
Source-drain current-voltage (*I*_DS_-*V*_DS_) characteristics of a MoS_2_-based FET (**a**) at *T* = 300 K as a function of back gate voltage (*V*_g_), (**b**) at *T* = 10 K as a function of *V*_g_, (**c**) at *V*_g_ = 0 V as a function of temperature, and (**d**) at *V*_g_ = 20 V as a function of temperature. The inset in (**a**) shows *I*_DS_-*V*_DS_ characteristics under *V*_DS_ of range ±0.1*V*_._ The arrow in (**c**,**d**) shows the direction of increase of *I*_DS_ with increasing temperature.

To understand the electrical behavior of the Py/MoS_2_ contacts, we carried out systematic two-probe *I*_DS_-*V*_DS_ curves measurements as a function of *V*_g_ and temperature for device #A1, and the results are shown in Fig. 2. Figure 2(a) shows *I*_DS_-*V*_DS_ characteristics at 300 K as a function *V*_g_. The *I*_DS_-*V*_DS_ curves are linear and symmetric under a small range (±0.1*V*) of *V*_DS_ (inset figure), however show small deviation from linearity and asymmetric behavior under high range (±1*V*) of *V*_DS_ (main figure). *I*_DS_-*V*_DS_ curves measured for device #A2 are almost linear and symmetric even under high range (±1*V*) of *V*_DS_ (see Supplementary Fig. S1), which reflects better device quality (we note that this device was unfortunately broken during the temperature dependent measurements). It can also be noted from Fig. 2(a) that *I*_DS_ increases with increasing *V*_g_—for a fixed applied *V*_DS_, at *V*_g_ = 20 V, obtained *I*_DS_ is at least 15 times higher than that of *V*_g_ = 0 V. It shows the tunability of FET characteristics with the application of *V*_g_ and suggests that the Schottky barrier is modified at the Py/MoS_2_ interface, which can result in the reduction of the SBH. As temperature is lowered, *I*_DS_-*V*_DS_ characteristics show strong deviation from linearity and significant reduction in *I*_DS_, as can be seen in Fig. 2(b). This suggests that the device goes in the off state at low temperatures.

Figure 2(c,d) show *I*_DS_-*V*_DS_ characteristics as a function of temperature at *V*_g_ of 0 V and 20 V, respectively. The arrows in these figures show the direction of increase of *I*_DS_ with increasing temperature. At fixed temperature and *V*_DS_, the *I*_DS_ is higher at *V*_g_ = 20 V than *V*_g_ = 0 V. Indeed, the Schottky barrier is lowered at 20 V, therefore more carriers can be thermally activated and overcome the barrier.

SBH can be extracted using an activation energy method, the commonly used one. The advantage of the activation energy method is that we do not need information of electrically active area under the contacts to extract the SBH^25^. The SBH for 2D materials can be extracted by employing the 2D thermionic emission equation^26,27^,

1 $${I}_{DS}=A{A}^{\ast }{T}^{\frac{3}{2}}\exp [-\frac{q}{{k}_{B}T}({\varphi }_{B}-\frac{{V}_{DS}}{n})],$$

where *A* is the contact surface area, A* is the effective Richardson constant, *q* is the electronic charge, *k*_B_ is the Boltzmann constant, *ϕ*_*B*_ is the Schottky barrier height and *V*_DS_ is source-drain voltage, and *n* is the ideality factor. From above equation, the activation energy is given by ${E}_{A}=q({\varphi }_{B}-\frac{{V}_{DS}}{n})$. After rearranging few terms, the equation can be written as

2 $$ln({I}_{DS}/{T}^{\frac{3}{2}})=\,\mathrm{ln}\,A+ln{A}^{\ast }-\frac{{E}_{A}}{{k}_{B}}(\frac{1}{T})$$

From Eq. (2), it is clear that *E*_A_ can be estimated from the slope of $ln({I}_{DS}/{T}^{\frac{3}{2}})$ vs. 1/*T*, called the Arrhenius plot. Once *E*_A_ is estimated, the SBH (*ϕ*_*B*_) can be extracted by simply taking the intercept of *E*_A_ vs. *V*_DS_ plot, which takes into account an effect of band bending of MoS_2_ by an application of the source-drain voltage.

**Figure 3:**
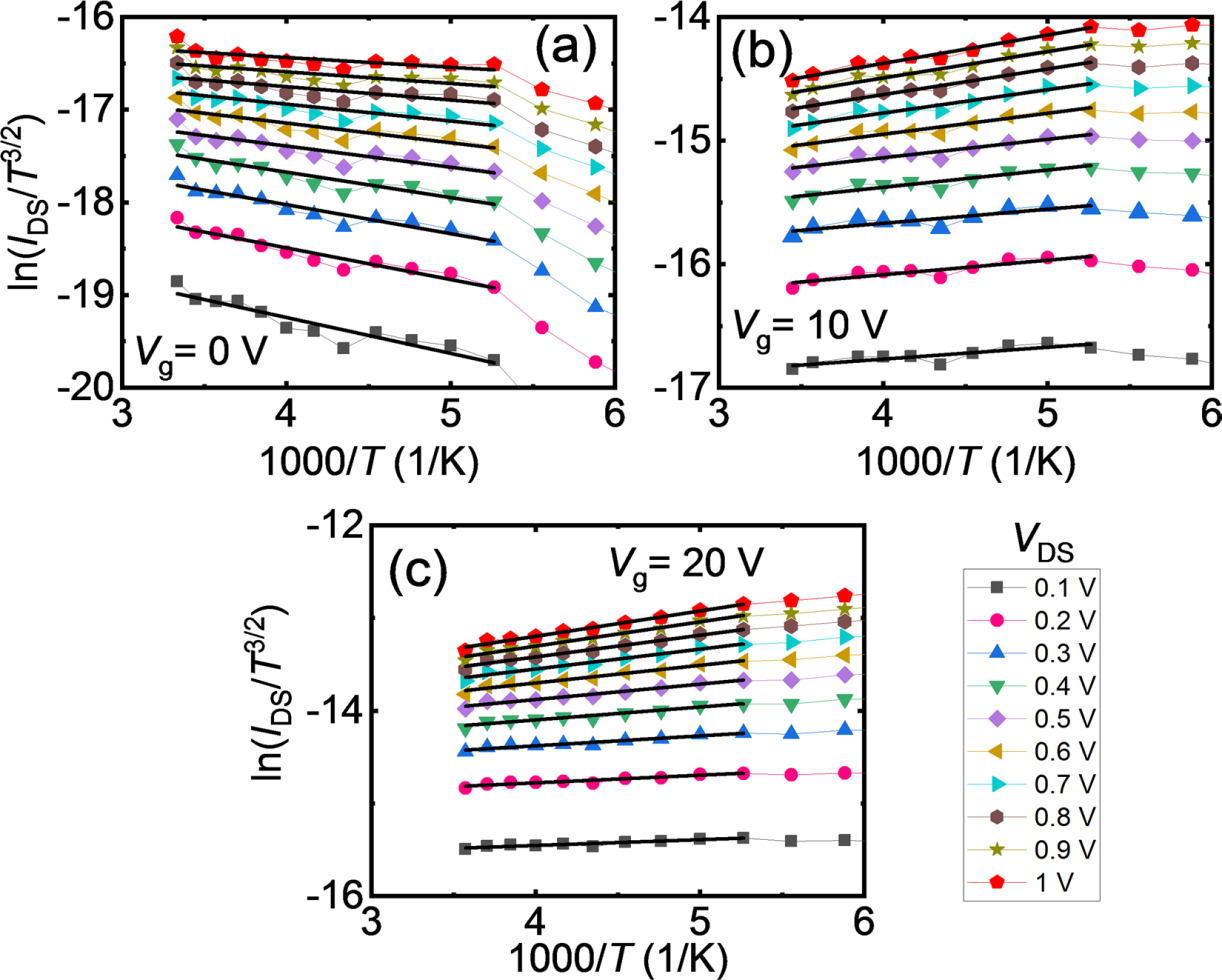
Arrhenius plots, $ln({I}_{DS}/{T}^{\frac{3}{2}})$ vs. 1000/*T* as a function of source-drain voltage (*V*_DS_) for different back gate voltages, (**a**) *V*_g_ = 0 V, (**b**) *V*_g_ = 10 V, and (**c**) *V*_g_ = 20 V. The solid black line is a linear fit to Arrhenius plot to extract the slope.

*I*_DS_-*V*_DS_ curves recorded in high temperature range (290 −190 K) were employed to calculate the SBH. At low temperatures, the device remains in off state because the thermal energy supplied to carriers is not enough to overcome the barrier and therefore the thermionic emission theory cannot be applied successfully. Figure 3(a–c) show Arrhenius plots for various *V*_DS_ at different *V*_g_. To determine the slope of the plot, the experimental data were fitted by Eq. (2) as shown in Fig. 3 as solid black lines. It is worth to note that the slope of the Arrhenius plot for *V*_g_ = 0 V is negative [Fig. 3(a)] and changes its sign from negative to positive with the application of *V*_g_ [Fig. 3(b,c)]. This indicates that the SBH decreases from a positive value (at *V*_g_ = 0 V) to negative values for *V*_g_ = 10 V and 20 V.

**Figure 4:**
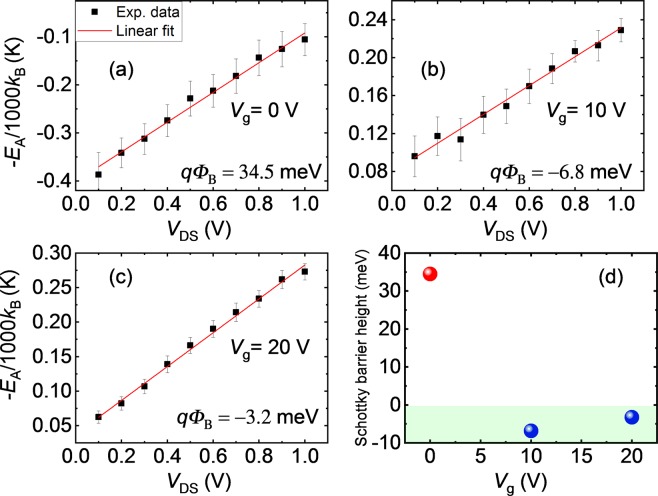
Source-drain voltage (*V*_DS_) dependence of slopes (−*E*_A_/1000*k*_B_) extracted from Fig. 3 for (**a**) back gate voltages, *V*_g_ = 0 V, (**b**) *V*_g_ = 10 V and (**c**) *V*_g_ = 20 V. The solid red line is a linear fit to the experimental data to extract the intercept and therefore Schottky barrier height (*ϕ*_*B*_). (**d**) *V*_g_ dependence of Schottky barrier height, showing positive value for *V*_g_ = 0 V and negative values for *V*_g_ = 10 and 20 V.

The slope of the Arrhenius plot as a function of *V*_DS_ is depicted in Fig. 4 for different *V*_g_ and fitted by linear function. The intercept from the linear fit gives the value of SBH, which is found to be +34.5 meV for *V*_g_ = 0 V. The SBH estimated for *V*_g_ = 10 and 20 V from the intercept in Fig. 4(b,c) are found to be −6.8 and −3.2 meV, respectively. To confirm the reproducibility of our results, we also estimated SBH for other FET devices fabricated on different samples. The SBHs estimated for devices #B1, #B2 and #C1 were found to be +26.0, +24.6 and 30.1 meV, respectively (see Supplementary Material), which strengthens the central claim of this study. It is quite important to estimate the SBH in TMD-based devices for future spintronic applications, because formation of the Schottky barrier strongly hinders efficient spin injection and spin detection. This is the reason we have been focusing on the SBH of the Py/MoS_2_ by modulating the source-drain and gate voltages, and indeed, it is significant that we have clarified appearance of the approximately zero barrier height. Meanwhile, in a fundamental point of view, it is also important to estimate SBH at flat band gate voltage. Hence, we fabricated device #C1 and measured both the zero- gate voltage and the flat-band SBH in the same device. As can be noted from Fig. S4 in the supplementary Material that the zero- gate voltage and flat band SBHs are 30.1 and 21.8 meV, respectively. The observed SBH value of +28.8 meV (average value) at *V*_g_ = 0 V is 64% lower than the previously reported SBH in Py/MoS_2_ contacts^16^. The small zero gate bias SBH observed in our devices indicates the ohmic nature of Py/MoS_2_ contacts. In the previous reports the SBH for ferromagnetic contacts such as Py/MoS_2_ and Co/MoS_2_ was reported to be 80.2 and 60.6 meV, where in the first case, the authors fabricated FET devices after transferring CVD grown MoS_2_ to SiO_2_/Si substrate via PMMA stamping method^16^ and in the latter case, the FET devices were fabricated using the MoS_2_ channels exfoliated from bulk MoS_2_ crystals^13^. The values of SBH estimated in previous reports for various FM and non-magnetic (NM) metal contacts on monolayer MoS_2_ channel are compared in Table 1. From the Table 1, it is clear that the observed SBH in our study is the smallest value reported so-far in any direct FM (non-magnetic)/monolayer MoS_2_ contact. The best value reported previously for direct metal/monolayer MoS_2_ contact, patterned by EB lithography was ~38 meV^12,28^. In fact, the SBH becomes small for transferred Ag electrodes with multilayer MoS_2_ channels^29^. However, multilayer MoS_2_ is not a direct band-gap semiconductor and spin-valley locking effect is somewhat suppressed^30^. Since one of the purposes of our study is exploring a potential of combination of ferromagnet and monolayer TMDs in spintronics viewpoints, realization of low SBH in Py/monolayer MoS_2_ is crucial, although the low SBH formation to a multilayer TMD is also notable. It can be noted that MoS_2_ channel used in previous reports were either exfoliated and/or transferred from CVD grown MoS_2_. This implies that FET devices fabricated using MoS_2_ directly grown on substrate via CVD technique decreases the chance of introducing distortion-induced defects during the exfoliation or the transferring methods and/or that of surface contamination, unlike exfoliated and transferred MoS_2_ techniques. It is worth to recall that in our case MoS_2_ is grown by salt-assisted CVD technique, which shows excellent crystal quality over exfoliated and CVD MoS_2_ as demonstrated by the smallest SBH reported so-far in exfoliated and/or CVD MoS_2_. High performance devices fabricated on directly grown CVD MoS_2_ confirms the possibility of mass production of MoS_2_-based devices. As aforementioned, circumventing the formation of the Schottky barrier is quite significant to realize efficient spin injection and detection in FM/semiconductor heterostructure, our results demonstrate the importance of an integration of directly grown MoS_2_ channels.

**Table 1:** Comparison of Schottky barrier heights (SBHs) for direct metal (magnetic and non-magnetic) contact on monolayer MoS_2_ channel.

Metal contact	MoS_2_ nature	SBH (meV)	Reference
Ni_80_Fe_20_ (Py)	Monolayer, directly grown CVD	device #A1 = 34.5 device #B1 = 26.0 device #B2 = 24.6 device #C1 = 30.1 (flat band = 21.8) **Average = 28.8**	This work
Ni_80_Fe_20_ (Py)	Monolayer, transferred CVD	80.2	^16^
Co	Monolayer, Exfoliated	60.6	^13^
Co	Monolayer, Exfoliated	~38	^12^
Ti	Monolayer, Exfoliated	230	^31^
Cr	Monolayer, Exfoliated	130	^31^
Au	Monolayer, Exfoliated	320	^31^
Pd	Monolayer, Exfoliated	300	^31^

## Conclusions

In conclusion, we fabricated FET devices using directly grown monolayer MoS_2_ channels on SiO_2_/Si substrate via salt-assisted CVD technique. The electrical properties of FET devices were studied by measuring two-probe *I*-*V* characteristic as a function of temperature and back gate voltage. The SBH estimated at *V*_g_ = 0 is found to be +28.8 meV, which is the smallest SBH reported so-far for any direct ferromagnetic as well as non-magnetic metal contact on monolayer MoS_2_. The small zero- gate voltage SBH indicates ohmic Py/MoS_2_ contacts. Ferromagnetic contacts with the smallest SBH and controllable ohmic contacts studied in the present study can open a route for practical realization of high performance MoS_2_ based spintronic devices.

## Supplementary information

Supplementary Information

## Data Availability

Data measured or analyzed during this study are available from the corresponding author on request.
