# Supplementary material for: Monolayer MoS2 field effect transistor with low Schottky barrier height with ferromagnetic metal contacts
Source: Sci Rep. 2019 Nov 19;9:17032. doi: 10.1038/s41598-019-53367-z (PMC6863907; doi:10.1038/s41598-019-53367-z)
Supplement: Supplementary file 1 — Supplementary Information [file 41598_2019_53367_MOESM1_ESM.pdf]

Supplementary Material

Monolayer MoS_2 field effect transistor with low Schottky barrier height with ferromagnetic metal contacts

Sachin Gupta,[1,*] F. Rortais,[1] R. Ohshima,[1] Y. Ando,[1] T. Endo,[2] Y. Miyata,[2] and M. Shiraishi[1]

[1]Department of Electronic Science and Engineering, Kyoto University, Kyoto, Kyoto 615-8510, Japan

[2]Department of Physics, Tokyo Metropolitan University, Hachioji 192-0397, Tokyo, Japan

*E-mail: gupta.sachin.2e@kyoto-u.ac.jp

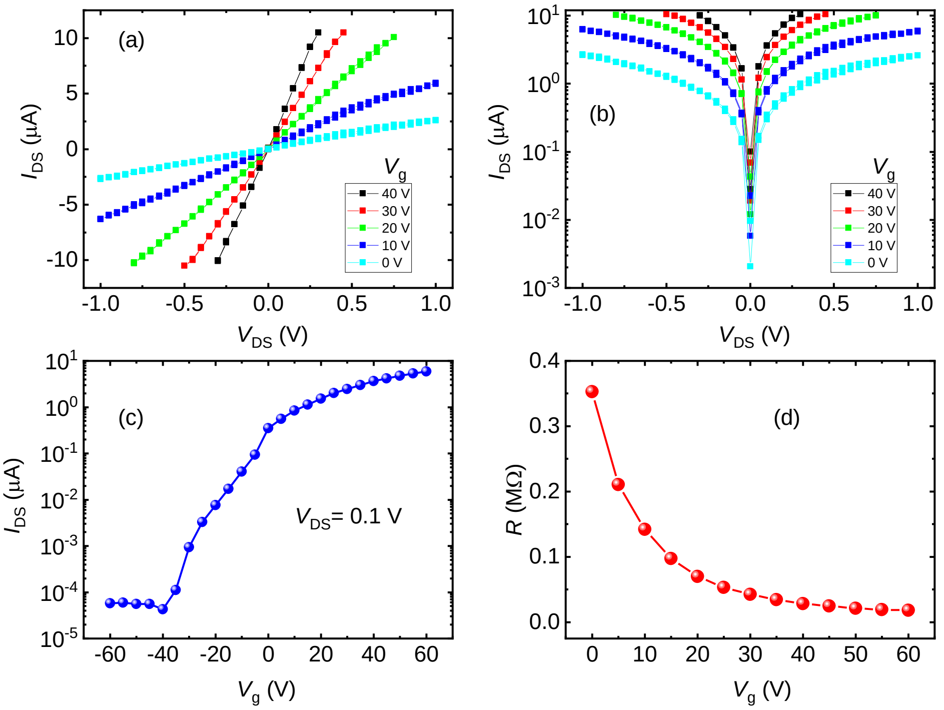

Figure S1. Room temperature characteristics of the FET device #A2: Source-drain current-voltage (I_{DS}-V_{DS}) characteristics of the FET plotted (a) on linear scale, (b) on semi-logarithmic scale as a function of back gate voltage (V_g). (c) V_g dependence of I_{DS} at V_{DS} = 0.1 V, plotted on semi- logarithmic scale. (d) V_g dependence of zero bias contact resistance (R).

Figure S1 shows the room temperature characteristics of the device #A2. Source-drain current-voltage (I_{DS}-V_{DS}) characteristics as a function of back gate voltage (V_g) are shown in Figure S1(a). It can be seen that I_{DS}-V_{DS} are linear and symmetric, suggesting ohmic contacts for this device. Figure S1(b) shows the same I_{DS}-V_{DS} curves plotted on semi-logarithmic scale. The V_g dependence of I_{DS} at V_{DS} = 0.1 V is plotted in Figure S1(c). I_{DS} increases with increasing V_g and shows an on/off ratio ~10^5 at V_{DS} = 0.1 V. Figure S1(d) shows V_g dependence of two-probe resistance (R). The R shown here has two contributions: two contact resistances and the channel resistance. The R decreases with V_g and shows a saturation trend at V_g= 60V.

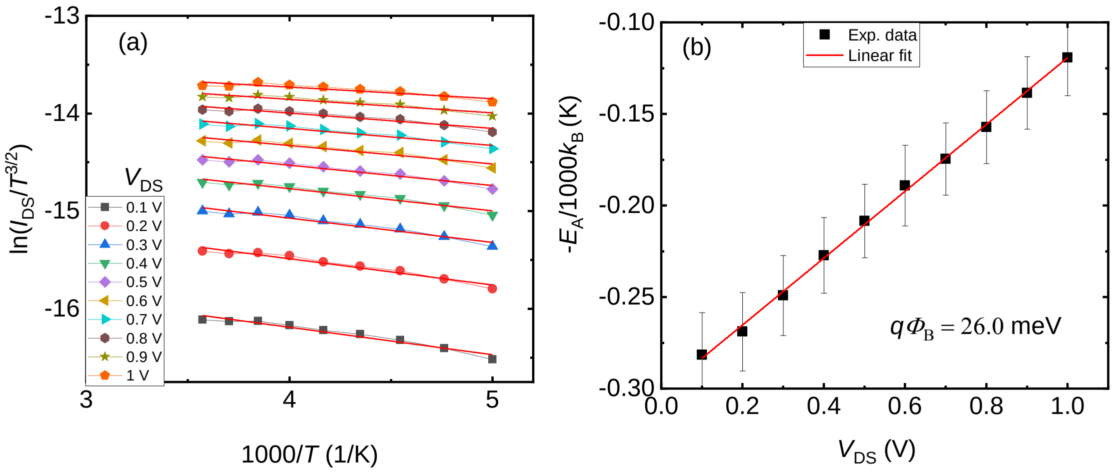

Figure S2 Schottky barrier height calculation for the FET device #B1 at V_g= 0 V: (a) The Arrhenius plot, $ln\left(I_{DS}/\,T^{\frac{3}{2}}\right)$ vs. 1000/T as a function of source-drain voltages (V_{DS}). (b) Source-drain voltage **(**V_{DS}) dependence of slopes (-E_A/1000k_B) extracted from (a) The solid lines are linear fits to the experimental data to extract the slope and intercept.

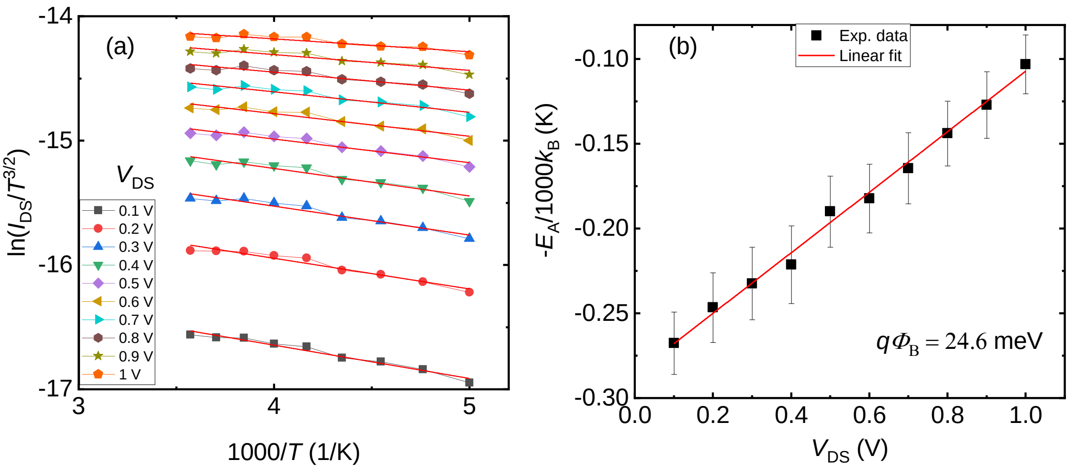

Figure S3 Schottky barrier height calculation for the FET device #B2 at V_g= 0 V: (a) The Arrhenius plot, $ln\left(I_{DS}/\ T^{\frac{3}{2}}\right)$ vs. 1000/T as a function of source-drain voltage (V_{DS}). (b) Source-drain voltage **(**V_{DS}) dependence of slopes (-E_A/1000k_B) extracted from (a) The solid lines are linear fits to the experimental data to extract the slope and intercept.

Figures S2(a) and S3(a) show Arrhenius plots, $ln\left(I_{DS}/\ T^{\frac{3}{2}}\right)$ vs. 1000/T as a function of source-drain voltages (V_{DS}) for the devices #B1 and #B2, respectively, and figures S2(b) and S3(b) show source-drain voltage **(**V_{DS}) dependence of slopes (-E_A/1000k_B) for the devices #B1 and #B2. The procedure for estimating Schottky barrier height (SBH) is described in main text. The SBHs estimated from the devices #B1 and #B2 at V_g= 0 V, are found to be 26.0 and 24.6 meV, respectively.

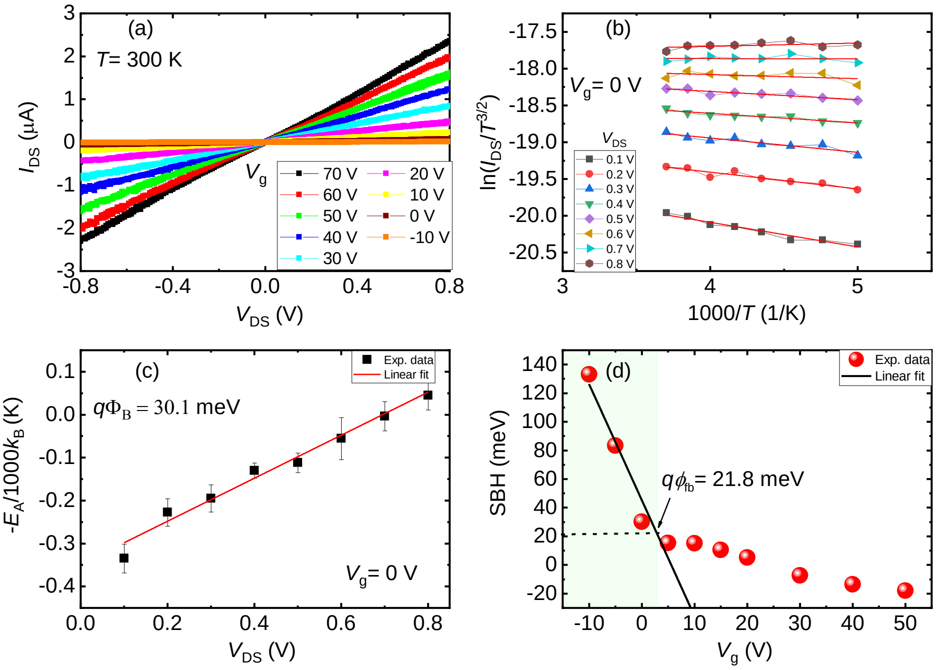

Figure S4 Schottky barrier height (SBH) calculation for the FET device #C1 at various V_g: (a) Room temperature source-drain current-voltage (I_{DS}-V_{DS}) characteristics of the FET at various V_g. (b) The Arrhenius plot, $ln\left(I_{DS}/\,T^{\frac{3}{2}}\right)$ vs. 1000/T as a function of source-drain voltage (V_{DS}). (c) Source-drain voltage **(**V_{DS}) dependence of slopes (-E_A/1000k_B) extracted from (b). The solid lines are linear fits to the experimental data to extract the slope and intercept. (d) V_g dependence of SBH to extract flat band SBH.

Figure S4 shows the FET characteristics for device #C1. To calculate the SBH, we measured I_{DS}-V_{DS} curves as a function of temperature and V_g. Figure S4(a) shows I_{DS}-V_{DS} curves as a function of V_g at room temperature. It can be noted that I_{DS}-V_{DS} curves are almost linear. Figure S4(b) shows Arrhenius plots, $ln\left(I_{DS}/\,T^{\frac{3}{2}}\right)$ vs. 1000/T as a function of source-drain voltages (V_{DS}). It is worth to note that with increasing $V_{DS,}$ the slope changes from negative to positive, which can be clearly seen in Fig. S4(c). We plotted activation energy as a function of V_{DS} and fitted the data by linear function to extract SBH. The SBH for device #C1 is estimated to be 30.1 meV at zero-bias, which is in the same

range as estimated for other devices. Figure S4(d) shows V_g dependence of SBH. The flat-band SBH estimated using linear fit is found to be 21.8 meV.

The SBHs estimated for all four devices are almost the same, which shows consistency and reproducibility of our results and indicates that devices fabricated using MoS_2 channels directly grown on the substrate via CVD technique can result in low SBH due to less defects and/or contamination as compared to transferred or exfoliated MoS_2 as discussed in the main text.

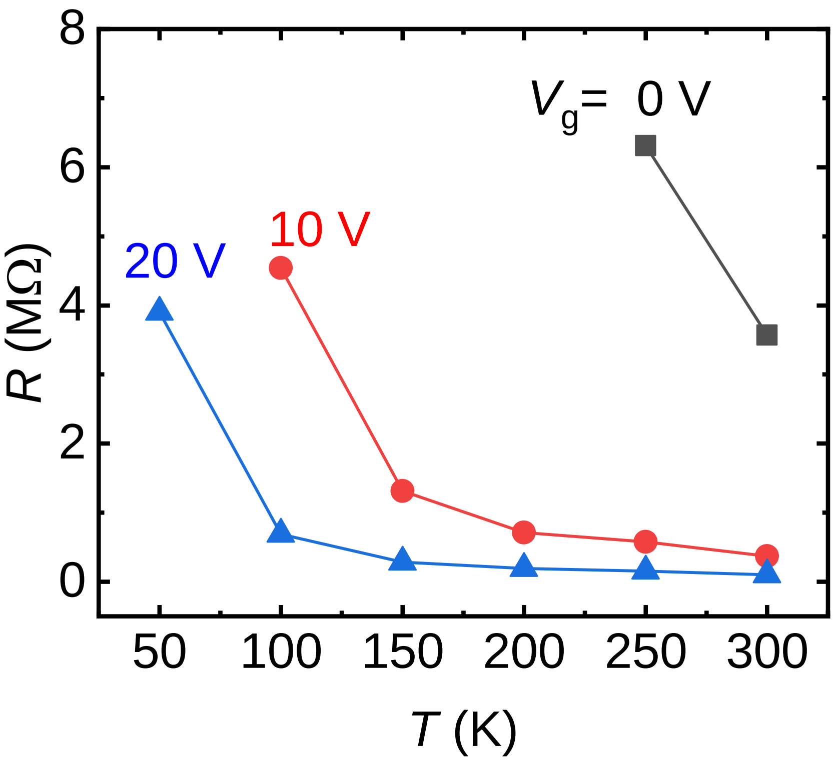

Figure S5. The temperature (T) dependence of zero-bias two-probe resistance (R) as a function of back gate voltage (V_g).

Figure S5 shows the temperature dependence of two-probe resistance (R) as a function of V_g. The R shown here has two contributions: two contact resistances and the channel resistance. One can note that at V_g= 0 V, the R is of the order of few MΩ and increases with decreasing temperature. At the low temperatures, the resistance exceeds 100 MΩ as the device goes in the off state. The R falls off abruptly with an application of V_g and is almost constant (in high temperature regime), which reflects ohmic behavior of

the contacts, resulting from low SBH. The R decreases with increasing V_g positively, which indicates the n-type behavior of FET devices as expected.
